# Significance of monitoring plasma concentration of voriconazole in a patient with liver failure: A case report

**DOI:** 10.1097/MD.0000000000008039

**Published:** 2017-10-20

**Authors:** Xiaoyan Liu, Haibin Su, Jingjing Tong, Jing Chen, Haozhen Yang, Long Xiao, Jinhua Hu, Lina zhang

**Affiliations:** Liver Failure Treatment and Research Center, 302 Hospital of PLA, Xisihuanzhonglu, Beijing, China.

**Keywords:** fungal pneumonia, liver failure, plasma concentration, voriconazole

## Abstract

**Rationale:**

Invasive pulmonary aspergillosis is associated with significant morbidity and mortality in patients with liver failure. Voriconazole (VRCZ) is recommended as a primary therapeutic agent for the treatment of invasive aspergillosis and metabolized in the liver. Now, data are still lacking on the safety and appropriate dosage of VRCZ in patients with liver failure. Here, we report a representative case of invasive pulmonary fungal infection in a patient with liver failure who was treated with low-dose VRCZ.

**Patient concerns:**

A 21-year-old man, presented with subacute liver failure caused suspected by viral infection, was admitted on June 22, 2014. Liver function was not improved by the treatment of gancicolovir and methylprednisolone. The patient presented with fever, cough, and hyperpyrexia on July 14. Laboratory tests revealed raised neutrophil percentage (82.1%, normal range [NR] 50–70), international normalized ratio (INR) (2.32, NR 0.8–1.2) and levels of serum lactic acid (4.308 mmol/L, NR 0.6–2.2), alanine transaminase (165 U/L,NR 0–40), aspartate transaminase (99 U/L, NR 8–40), and total bilirubin (654 mmol/L, NR 3.4–20.5). Furthermore, CD4+ T cell, CD8+T cell, and B cell count were low (169, 221, and l8/mL, respectively). Sputum smear microscopy for bacteria was negative, but the direct observation for fungal elements was positive. Thoracic CT scan revealed bilateral pulmonary high-density shadow. Sputum cultures were positive 2 days later with the presence of Aspergillus fumigatus.

**Diagnoses:**

Therefore, this patient diagnosed with suspected pulmonary a spergillosis.

**Interventions:**

VRCZ was used on July 15th and its dosage was 400 mg twice on day 1 followed by a maintenance dose of 100 mg twice daily according to drug usage instruction. However, some side effects, such as tremors, lips twitching, and hair loss, occurred. Plasma VRCZ trough concentration was 8.1 mg/mL which was much higher than the recommend level. Therefore, VRCZ dosage was adjusted according to its plasma concentration. VRCZ plasma concentration fluctuated between 2.5 to 4.7 mg/mL when its dosage was 100 mg once daily and side effects disappeared.

**Outcomes:**

VRCZ was administered for 2 months. This patient's symptoms and liver function were improved. A follow-up CT scan performed at the end of VRCZ therapy indicated that the high-density shadow had diminished.

**Lessons:**

This case demonstrated that low-dose VRCZ (maintenance dose, 100 mg/day) can achieve effective plasma concentration and reduce side effects without liver damage. We believe that VRCZ is safe to be administered in patients with liver failure, but its plasma concentration should be carefully monitored.

## 1 Introduction

Liver failure is generally characterized by acute deterioration of liver function. Due to secondary immunodeficiency and long-term use of antibiotics, patients with liver failure are prone to infections.^[1]^ Invasive fungal infections (IFIs), particularly invasive pulmonary aspergillosis, in patients with liver failure is associated with significant morbidity and mortality.^[2]^ According to the guidelines published by the Infectious Diseases Society of America, early initiation of antifungal therapy in patients with strongly suspected invasive aspergillosis is warranted while conducting a diagnostic evaluation.^[3]^ Generally, empirical and preemptive antifungal therapy should be used immediately after pulmonary aspergillosis is suspected in patients with liver failure who have a high risk for fungal infection, including evidence of fever, history of long-term antibiotic use, fungal positive in sputum culture, and plaques on computed tomography (CT) imaging.

The drug is metabolized in the liver, at the level of P450 (CYP2C19, CYP2C9, and CYP3A4), and the products of metabolism are excreted by the kidneys.^[3]^ Therapeutic and adverse events are usually closely related to the drug's plasma concentration.^[4]^ Drug plasma concentration monitoring can help physician adjust the dose of the antifungal therapy and achieve good results while reducing, or even avoiding unnecessary adverse events. The medication label of voriaconazole (VRCZ) indicates that drug metabolism was in disorder and that the dose should be halved in patients with liver disease, such as in patients with cirrhosis and mild-to-moderate liver dysfunction (Child–Pugh A or B). However, data are still lacking on the safety and appropriate dosage of VRCZ in patients with severe liver dysfunction (Child–Pugh C) or liver failure. Here, we report a representative case of invasive pulmonary fungal infection in a patient with liver failure who was treated with low-dose VRCZ. We demonstrated that even low-dose VRCZ can achieve effective plasma concentration without severe adverse events.

## 2 Case report

A 21-year-old man, presented with subacute liver failure caused suspected by viral infection, was admitted on June 22, 2014, with intermittent fever, a maximum body temperature of 39 °C, and hyperbilirubinemia for 20 days. He had no history of alcohol and drug abuse. The results of blood tests at admission are as follows: total bilirubin (TBIL), 389 μmol/L; alanine aminotransferase, 254 U/L; aspartate aminotransferase, 314 U/L; and INR, 1.65; serum copper and ceruloplasmin levels were within NR. Routine blood tests were normal. Serum markers for hepatitis A, B, C, and E, anti-EBV IgM, anti-CMV IgM, anti-HIVIgM, and autoantibodies were negative. He was diagnosed with subacute liver failure of unknown etiology. After admission, the patient was treated with ganciclovir on June 29, because we suspected he had a viral infection based on the following findings: enlarged lymph nodes and fever, and ruled out other causes of bacterial infection. Methylprednisolone was used concurrently to inhibit systematic inflammation reaction. Body temperature returned to normal on the second day after the initiation of therapy. However, liver function deteriorated within 10 days of starting therapy; TBIL increased to 600 μmol/L and INR increased to 2.6. Therefore, ganciclovir was stopped on July 14th because it was thought to not be effective. His model for end-stage liver disease (MELD) score was 32.9 (Table 1). He then developed worsening fever and cough for which a sputum culture was obtained with fungal culture positive at 2 days with *Aspergillus fumigatus* that was VRCZ susceptible. Subsequent thoracic CT scan revealed bilateral pulmonary high-density shadow and we suspected that the patient had invasive aspergillosis. Considering the severity of the patient's condition and since we had a high suspicion that he had fungal pneumonia, we treated him VRCZ on July 15. Methylprednisolone dose was reduced gradually and stopped on July 27. Oral VRCZ 400 mg twice on day 1, followed by a maintenance dose of 100 mg twice daily was administered according to drug usage instruction. However, this patient began to have tremors, lips twitching, and hair loss on July 27th. Plasma VRCZ trough concentration was measured according to the protocol used in other studies.^[5,6]^ VRCZ plasma concentration was 8.1 μg/mL on July 28, which was much higher than the recommend level.^[7,8]^ Therefore, VRCZ dose was reduced to 100 mg once daily. VRCZ trough concentration decreased to 7 μg/mL on July 31, but the patient's symptoms had not improved. VRCZ was stopped on August 1st, and we began to administer 100 mg every other day on August second. The patient's symptoms disappeared on August 3. VRCZ trough concentration (2 hours before VRCZ administration) decreased to 5.2 μg/mL on August 4th, without any side effects. These symptoms were considered to be caused by high plasma concentration of VRCZ. Because VRCZ trough concentration was 1.2 μg/mL on August 8, which is lower than the recommend level, VRCZ dose was increased to 100 mg once daily until the end of VRCZ treatment on September 18th. VRCZ trough concentration fluctuated between 2.5 and 4.7 μg/mL. The study protocol was approved by the Ethics Committee at the 302 Hospital. A written informed consent was systematically obtained.

**Table 1:**
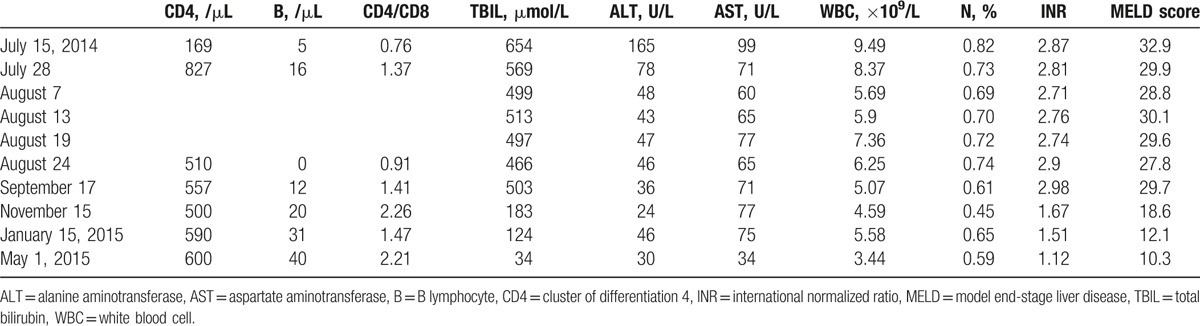
Laboratory findings during the treatment.

**Figure 1:**
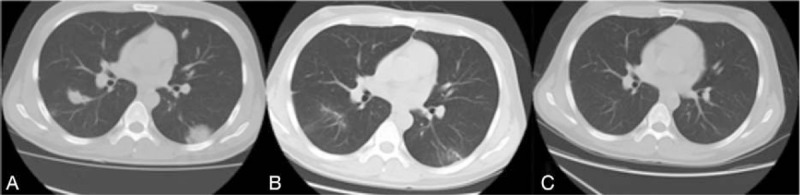
Chest computed tomography (CT) scan at baseline (A), 2 weeks (B), and 2 months after treatment (C), indicating the disappearance of pulmonary consolidation.

A follow-up CT scan performed at the end of VRCZ therapy indicated that the high-density shadow had diminished (Fig. 1). Liver function was improved: TBIL and INR decreased to 499 μmol/L and 1.9, respectively, during antifungal therapy. The total VRCZ treatment duration was 60 days. In addition, it should be noted that the patient developed severe spontaneous bacterial peritonitis and hepatic encephalopathy during treatment, that is, on September 28th and October 3rd, respectively. The patient was discharged on November 15th and 2 months later, follow-up results showed stable liver function and normal CT imaging findings (Table 1).

## 3 Discussion

*A fumigatus* is typically susceptible to VRCZ. VRCZ is a 2nd-generation broad-spectrum triazole antifungal agent that is currently being used to treat a wide variety of fungal infections.^[9,10]^ It depletes ergosterol and suppresses fungal cell growth and replication. It has been recommended as a primary therapeutic agent for invasive aspergillosis and is available orally and intravenously. Clinical outcomes and adverse events are related to VRCZ concentration. Dolton et al^[4]^ reported that VRCZ concentrations <1.7 μg/mL are associated with a significantly greater incidence of treatment failure and that neurotoxic adverse events, including visual and auditory hallucinations, occurred more frequently with concentrations >5 μg/mL. Therefore, monitoring plasma concentrations of VRCZ is essential.

Adverse events of VRCZ commonly include abdominal pain, elevated transaminase levels, fever, nausea, phototoxicity, and vision abnormalities. Research papers on this subject have disparate assessments. One study showed that adverse symptoms occurred in 21% of patients with acute myelogenous leukemia or high-risk myelodysplastic syndrome taking VRCZ.^[11]^ Another study reported that two-thirds of patients treated with VRCZ had severe reactions to the drug, including elevated transaminases in 35%, cholestasis in 15%, or a combination of both in 45% of patients.^[12]^ The most common clinical feature was the new onset jaundice (41.7%) and skin irritation (41.7%). Several studies also demonstrated that VRCZ plasma concentration was closely associated with treatment efficacy and side effects.^[12–17]^ In one study, researchers concluded that the risk for phototoxic skin reactions were associated with dosage of VRCZ.^[18]^ A meta-analysis conducted by Hamada et al^[19]^ indicated that the success rate for fungal infection treatment increased significantly with VRCZ levels >1.0 μg/mL when a graded cutoff value was within the range of 1.0 to 3.0 μg/mL was used as the standard VRCZ trough plasma concentration. However, the incidence of adverse neurological events increased with increasing VRCZ plasma concentration. They suggested that for safety reasons, VRCZ trough blood concentration should be >1.0 μg/mL from the perspective of efficacy and <4.0 μg/mL. In another meta-analysis, researchers showed that a VRCZ level of 0.5 mg/L should be considered the lower threshold associated with efficacy. A trough concentration of 3.0 mg/L is associated with increased hepatotoxicity, particularly in the Asian population, and 4.0 mg/L is associated with increased neurotoxicity.^[20]^ Blood concentration of VRCZ can be affected by several factors, including patients’ age, race, drug–drug interactions, route of administration, and cytochrome P450 (CYP) polymorphism, predominantly CYP2C19.^[17,21–27]^ VRCZ is mainly metabolized in the liver by cytochromes CYP2C9, CYP 3A4, and CYP2C19.^[28]^ Therefore, the capacity of VRCZ metabolism by the liver is attenuated in patients with liver failure. One study showed that the average VRCZ plasma concentration was 1.4 μg/mL (range, 0–14.3 μg/mL) when 6.1 mg/kg per 12 hour was used in patients without liver disease.^[4]^ However, VRCZ plasma concentration was 8.1 μg/mL in our patient, even at a dose of 1.78 mg/kg per 12 hours without using any other drugs that could affect VRCZ concentration. Furthermore, methylprednisolone was used from June 28 to the end of July, which may have decreased VRCZ plasma concentration. The manufacturer's information indicated that VRCZ plasma concentration can be affected by liver function. However, a retrospective study suggested that VRCZ concentrations are unpredictable, despite standardized dosing and that an appropriate concentration on one occasion cannot necessarily be extrapolated to future dosage calculation for administration even in the same patient.^[29]^

To our knowledge, there are currently no uniform standards of VRCZ dosage and no reports presenting its safety in patients with severe liver dysfunction (Child–Pugh C), or in patients with liver failure. The drug label information indicated that VRCZ liver toxicity manifested as elevated transaminases to mild jaundice, which occurred more commonly in patients with decompensated liver diseases, especially when the initial VRCZ initial dose was greater 300 mg. Multifactor analysis indicated that high plasma concentration was associated with liver toxicity, which can be reduced with a VRCZ trough level below 4 μg/mL. However, in one study, researchers argued that there was little value to monitoring VRCZ plasma concentration in patients with or without liver failure.^[30]^ Liver function monitoring was considered to be more important than VRCZ plasma concentration monitoring.

The maintenance dose in this patient was half of the recommended dose, according to the instruction for patients with Child–Pugh score A and B. In this case, however, plasma concentration was 8.1 μg/mL, which is much higher than the recommended plasma concentration. Furthermore, the patient presented with rare side effects including hand tremors, lip twitching, and hair loss. These side effects disappeared after the dose was adjusted, and the lung infection and liver function improved. Therefore, we argue that only monitoring liver function is not enough in patients with liver failure. This case demonstrates that patients with liver failure can have a higher VRCZ plasma concentration even if they receive a low dose of VRCZ, and can still have improved liver function following treatment. This is consistent with other studies indicating that VRCZ plasma concentration is highly variable and that the correlation between dose and levels is weak in adult patients. We believe that VRCZ trough concentration should be monitored every 3 days to maintain the VRCZ treatment efficacy and avoid adverse events in patients with liver failure until its trough concentration achieve stable level.

In conclusion, this case demonstrates that steroids are a risk fact for fungal infection in patients with liver failure. It is safe to administer VRCZ in patients with fungal pneumonia, but VRCZ plasma concentration should be carefully monitored. VRCZ plasma concentration should be maintained within 1–1.5 to 5–6 μg/mL, according to studies and guidelines. Side effects should be monitored closely if VRCZ plasma concentration monitoring is not available. Dosage should be reduced once side effects occur or liver function deteriorates. VRCZ plasma concentration monitoring was not a routine test in our hospital; therefore, we tested VRCZ plasma concentration only after side effects occurred. VRCZ dose should be optimized for a better therapeutic outcome and minimize side effects by monitoring VRCZ plasma concentration. Our case report has several limitations. First, fungal pneumonia was diagnosed without performing pathological examination. Second, cytochrome P450 polymorphism was not detected, which may have affected VRCZ metabolism. This case demonstrates that VRCZ can be used safely in patients with liver failure patients using dose-adjustments and that plasma concentration monitoring is necessary.

## References

[1] Philips CA Sarin SK Sepsis in cirrhosis: emerging concepts in pathogenesis, diagnosis and management. Hepatol Int 2016;10:871–82. 27422251 10.1007/s12072-016-9753-2

[2] Lin LN Zhu Y Che FB Invasive fungal infections secondary to acute-on-chronic liver failure: a retrospective study. Mycoses 2013;56:429–33. 23368965 10.1111/myc.12044

[3] Walsh TJ Anaissie EJ Denning DW Treatment of aspergillosis: clinical practice guidelines of the Infectious Diseases Society of America. Clin Infect Dis 2008;46:327–60. 18177225 10.1086/525258

[4] Dolton MJ Ray JE Chen SC-A Multicenter study of voriconazole pharmacokinetics and therapeutic drug monitoring. Antimicro Agents Chemother 2012;56:4739–99. 10.1128/AAC.00626-12 PMC3421881 22751544

[5] Khoschsorur GA Fruehwirth F Zelzer S Isocratic high-performance liquid chromatographic method with ultraviolet detection for simultaneous determination of levels of voriconazole and itraconazole and its hydroxy metabolite in human serum. Antimicrob Agents Chemother 2005;49:3569–71. 16048987 10.1128/AAC.49.8.3569-3571.2005 PMC1196257

[6] Langman LJ Boakye-Agyeman F Measurement of voriconazole in serum and plasma. Clinic Biochem 2007;40:1378–85. 10.1016/j.clinbiochem.2007.07.024 17931613

[7] Brüggemann RJ Donnelly JP Aarnoutse RE Therapeutic drug monitoring of voriconazole. Therap Drug Monit 2008;30:403–11. 18641555 10.1097/FTD.0b013e31817b1a95

[8] Trifilio S Update on antifungal drug dosing and therapeutic drug monitoring. Curr Fungal Infect Rep 2011;5:92–102.

[9] Sabo JA Abdel-Rahman SM Voriconazole: a new triazole antifungal. Ann Pharmacother 2000;34:1032–43. 10981251 10.1345/aph.19237

[10] Cecil JA Wenzel RP Voriconazole: a broad-spectrum triazole for the treatment of invasive fungal infections. Expert Rev Hematol 2009;2:237–54. 21082966 10.1586/ehm.09.13

[11] Mattiuzzi GN Cortes J Alvarado G Efficacy and safety of intravenous voriconazole and intravenous itraconazole for antifungal prophylaxis in patients with acute myelogenous leukemia or high-risk myelodysplastic syndrome. Support Care Cancer 2011;19:19–26. 19956980 10.1007/s00520-009-0783-3

[12] Solis-Munoz P Lopez JC Bernal W Voriconazole hepatotoxicity in severe liver dysfunction. J Infection 2013;66:80–6. 10.1016/j.jinf.2012.09.011 23041040

[13] Vöhringer S Schrum J Ott H Severe phototoxicity associated with long-term voriconazole treatment. J Dtsch Dermatol Ges 2010;9:274–6. 21050383 10.1111/j.1610-0387.2010.07563.x

[14] Okuda T Okuda A Watanabe N Retrospective serological tests for determining the optimal blood concentration of voriconazole for treating fungal infection. Yakugaku Zasshi 2008;128:1811–8. 19043301 10.1248/yakushi.128.1811

[15] Andes D Pascual A Marchetti O Antifungal therapeutic drug monitoring: established and emerging indications. Antimicrob Agents Chemother 2009;53:24–34. 18955533 10.1128/AAC.00705-08 PMC2612175

[16] Neely M Rushing T Kovacs A Voriconazole pharmacokinetics and pharmacodynamics in children. Clin Infect Dis 2010;50:27–36. 19951112 10.1086/648679 PMC2803104

[17] Pasqualotto AC Xavier MO Andreolla HF Voriconazole therapeutic drug monitoring: focus on safety. Expert Opin Drug Saf 2010;9:125–37. 20021293 10.1517/14740330903485637

[18] Bernhard S Kernland Lang K Roland A Voriconazole-induced phototoxicity in children. Pediatr Infect Dis J 2012;31:769–71. 22517339 10.1097/INF.0b013e3182566311

[19] Hamada Y Seto Y Yago K Investigation and threshold of optimum blood concentration of voriconazole: a descriptive statistical meta-analysis. J Infect Chemother 2012;18:501–7. 22231601 10.1007/s10156-011-0363-6

[20] Jin H Wang T Falcione BA Trough concentration of voriconazole and its relationship with efficacy and safety: a systematic review and meta-analysis. J Antimicrob Chemother 2016;71:1772–85. 26968880 10.1093/jac/dkw045 PMC4896404

[21] Kim DY Park HJ Lee YJ Factors affecting voriconazole plasma concentrations in patients with invasive fungal infections. Int J Clin Pharmacol Ther 2014;52:209–16. 24424111 10.5414/CP202014

[22] Weiss J Ten Hoevel MM Burhenne J CYP2C19 genotype is a major factor contributing to the highly variabl pharmacokinetics of voriconazole. J Clin Pharmacol 2009;49:196–204. 19033450 10.1177/0091270008327537

[23] Li X-Q Andersson TB Ahlström M Comparison of inhibitory effects of the proton pump-inhibiting drugs omeprazole, esomeprazole, lansoprazole, pantoprazole, and rabeprazole on human cytochrome P450 activities. Drug Metab Dispos 2014;32:821–7. 10.1124/dmd.32.8.821 15258107

[24] Scholz I Oberwittler H Riedel KD Pharmacokinetics, metabolism and bioavailability of the triazole antifungal agent voriconazole in relation to CYP2C19 genotype. Br J Clin Pharmacol 2009;68:906–15. 20002085 10.1111/j.1365-2125.2009.03534.x PMC2810802

[25] Heinz WJ Kloeser C Helle A Comparison of plasma trough concentrations of voriconazole in patients with or without co-medication of ranitidine or pantoprazole. Clin Microbiol Infect 2007;13 (Suppl 1):S357.

[26] van Wanrooy MJ Span LF Rodgers MG Inflammation is associated with voriconazole trough concentrations. Antimicrob Agents Chemother 2014;58:7098–101. 25223994 10.1128/AAC.03820-14 PMC4249508

[27] Saini L Seki JT Kumar D Serum voriconazole level variability in patients with hematological malignancies receiving voriconazole therapy. Can J Infect Dis Med Microbiol 2014;25:271–6. 25371690 10.1155/2014/214813 PMC4211351

[28] Niwa T Shiraga T Takagi A Effect of antifungal drugs on cytochrome P450 (CYP) 2C9, CYP2C19, and CYP3A4 activities in human liver microsomes. Biol Pharm Bull 2005;28:1805e8. 16141567 10.1248/bpb.28.1805

[29] Trifilio SM Yarnold PR Scheetz MH Serial plasma voriconazoleconcentrations after allogeneic hematopoietic stem cell transplantation. Antimicrob Agents Chemother 2009;53:1793–6. 19223632 10.1128/AAC.01316-08 PMC2681520

[30] Lutsar I Hodges MR Tomaszewski T Safety of voriconazole and dose individualization. Clin Infect Dis 2003;36:1087–8. 12684928 10.1086/374248

